# How Humanity Has Always Feared Change: Are You Afraid of Artificial Intelligence?

**DOI:** 10.7759/cureus.83602

**Published:** 2025-05-06

**Authors:** Mirella Veras

**Affiliations:** 1 Physical Therapy, University of Manitoba, Winnipeg, CAN

**Keywords:** artificial intelligence, artificial intelligence readiness, disruptive innovation, ethic, fear, history, public fear, societal equity, tech, technology evolution

## Abstract

This article explores the relationship between fear, technological progress, and human adaptation, focusing on the rise of artificial intelligence (AI). Throughout history, significant technological advancements have provoked excitement and apprehension, from the invention of writing to the Industrial Revolution. AI is the latest episode in this constant evolution, raising fears about job automation, ethical dilemmas, loss of creativity, and existential risks. However, like past innovations, AI also presents opportunities to revolutionize healthcare, productivity, and innovation. The question guiding this analysis is *How can societal fear of AI be understood not only as a reaction to disruption but also as a stimulus for ethical engagement and inclusive technological governance?* The article argues that fear is not inherently negative but instead a catalyst for reflection on the ethical implications of change. Drawing on historical examples and contemporary discourse, it highlights the importance of equity, ethics, and active engagement in technological development, encouraging society to influence the trajectory of AI. The article contributes to reframing fear as a constructive force that can guide critical inquiry, promote responsible technology innovation, and foster democratic participation in determining AI futures. Finally, it suggests that how we navigate AI’s rise will assess its role in society and its potential to redefine human progress. To move forward, the article advocates for proactive strategies that center equity, interdisciplinary dialogue, and ethical foresight in designing and deploying AI systems.

## Editorial

Fear as a constant companion to innovation

Change is rarely straightforward, particularly when it challenges the core of how we live, think, and work. Throughout history, each major technological shift has sparked a combination of excitement and fear. From the invention of writing to the industrial revolution, new tools and systems have forced humanity to reconsider its role in the world [1]. AI is no longer confined to science fiction or academic speculation. It is now an integral part of our daily lives [2], curating social media feeds, targeting personalized ads, and influencing the news and products that we encounter. Algorithms track our behavior, predict preferences, and subtly steer our decisions, shaping everything from online interactions to how we view the world. Time and again, major innovations have disrupted social norms while opening new possibilities. Such changes often trigger concerns about losing core aspects of human life, jobs, traditions, or autonomy, even as they unlock benefits. AI follows a similar path as earlier technological shifts, from the creation of writing to the Industrial Revolution and even modern developments such as the global positioning system (GPS), demonstrating humanity's complex relationship with change.

Today, AI represents a new chapter in our ongoing technological evolution. Like past innovations, it has ignited excitement over its potential and anxiety about its consequences. However, why does technological change often elicit fear? To understand this, we must look back to the early moments when humanity began reshaping how we communicate and share knowledge. However, AI's rapid rise is accompanied by questions of whether we should embrace it or fear it. Why does every significant technological breakthrough - from cuneiform writing to the industrial revolution - seem to keep both awe and anxiety? To understand why AI elicits such mixed reactions, we must first examine humanity's long history of confronting technologies and the fears they bring with them.

This article addresses the central question: How does fear shape society’s engagement with major technological change, and can it be reframed as a driver of ethical reflection and adaptation? Rather than treating fear solely as resistance, this analysis positions it as a historically recurrent and constructive response. This article will examine five major innovations and advancements: writing, GPS, calculators, statistical software, and the Industrial Revolution - and their impact on human cognition and society. These innovations, summarized in Table 1, represent milestones that not only changed how we think, work, and interact with the world but also sparked debates and concerns about their potential downsides. Through an analysis of their historical context, cognitive shifts, and societal implications, this discussion aims to highlight the balance between progress and the challenges each innovation brought to light.

**Table 1:** Major Innovation Milestones: Progress, Fears, and Cognition or Societal Impact Table Credits: Mirella Veras

Innovations	Historical Context	Impact on Cognition & Society	Connection to the Past/Future
1. Writing	Emergence of cuneiform (~3000 BCE)	Enhanced long-term knowledge preservation and communication. Shifted from oral traditions to written records.	Challenged memory, similar to AI tools expanding human knowledge.
2. GPS	Revolutionized navigation (late 20th century)	Reduced engagement with the environment; reliance on step-by-step directions leads to passive navigation.	Like writing, reshaped interaction with surroundings, creating new cognitive opportunities.
3. Calculators	Invention (1960s-1970s) allowed quick, complex computations	Reduced need for manual calculations; sparked debates about diminishing mental math skills.	Like the Industrial Revolution, streamlined tasks, impacting skills and labor.
4. Statistical Software	Development of tools like SPSS, R, Python for advanced analysis	Facilitated faster data processing but reduced understanding of underlying principles.	Automates processes, like calculators, statistical software, machine learning, reshaping cognitive demands in data analysis.
5. Industrial Revolution	Machines replaced manual labor in the 18th-19th centuries	Job displacement, urbanization, factory work, and changes in social structures.	Raised fears of human job replacement, akin to concerns about AI today.

The writing revolution: fear of memory loss

As outlined in Table 1, to understand the impact of AI, we must look back at its historical foundations, drawing parallels to the rise of writing. Just as the introduction of writing changed human cognition, AI presents similar debates about its potential to redesign our understanding of memory, intelligence, and creativity. In Phaedrus, Plato presents Socrates' argument that writing would weaken memory and diminish genuine understanding, as it promotes dependence on external symbols rather than internal reasoning. Philosophers such as Socrates feared that writing would weaken memory, and today, similar concerns arise about AI's effects on human cognitive abilities [3].

Over 5,000 years ago, the emergence of cuneiform writing transformed how humanity recorded and shared knowledge. However, even this monumental leap was met with skepticism and philosophical criticism [3]. Although Plato documented the critique through Socratic dialogue, the argument reflects enduring concerns about how external technologies might displace mental internal cognitive capacities [4]. These early philosophical perspectives, rooted in dialogue and observation, anticipated questions that modern cognitive science now explores through empirical research. While Socrates warned that writing could weaken memory and genuine understanding, today’s neuroscientists investigate how technology affects brain function, cognition, and reasoning. Similarly, today's discussion around AI focuses on whether increased reliance on intelligent systems could erode cognitive abilities such as memory, problem-solving, and critical thinking [2,5,6]. Concerns involve the potential decline in creativity and the risk of overdependence on AI, which could hinder individuals' capacity to question, evaluate, and make autonomous decisions [7].

Plato's concerns about writing diminishing memory and understanding parallel contemporary discussions about AI and its effects on human cognition. Just as the advent of writing reshaped how we share knowledge, AI challenges our traditional ideas about intelligence [4]. Alan Turing's 1950 prediction, where he stated, "I believe that at the end of the century, the use of words and general educated opinion will have altered so much that one will be able to speak of machines thinking without expecting to be contradicted," envisioned a time when machines might think like humans [8]. While we have not reached that point yet, Turing's insight remains a touchstone in the ongoing debate over AI's potential. Verba volant, scripta manent ("spoken words fly away, written words remain") - this Latin adage reminds us that, just as spoken thought was once fleeting, the written word and machines alike may persist in shaping our understanding of thought.

Recent advancements in neuroscience add a layer of complexity to the discussion, highlighting subtle distinctions that challenge conventional assumptions. Massachusetts Institute of Technology (MIT) neuroscientist Ev Fedorenko and her team have demonstrated through detailed experiments that language fluency is not directly linked to other cognitive abilities, such as reasoning or common sense [8]. This dissociation suggests that linguistic fluency does not inherently reflect broader cognitive abilities, such as critical thinking or problem-solving. These findings are particularly relevant in evaluating AI, which often exhibits high levels of language generation without corresponding evidence of general intelligence. The brain networks involved in language production are distinct from those responsible for critical thinking, suggesting that fluency in language does not equate to general intelligence [8]. Thus, we must reconsider what "thinking" truly means and whether AI's ability to generate language reflects a deeper form of intelligence or is merely a surface-level imitation of human behavior. Descartes' famous principle - cogito, ergo sum ("I think, therefore I am") - reminds us that true intelligence may not simply lie in fluency but in the more profound act of conscious thought [9]. As communication tools evolved, so did the technologies designed to extend human abilities. Turing's test, introduced in the mid-20th century, sought to determine whether a machine could replicate human-like thought, laying the groundwork for the AI we engage with today. Just as writing once sparked philosophical concerns, AI now invites similar scrutiny regarding its effects on human intellect and society.

The rise of writing led to the decline of oral traditions but also brought significant benefits. Writing allowed knowledge to be preserved over time, enabling the sharing of ideas and information in ways oral traditions could not. This ability to record and distribute knowledge became the foundation of modern civilization, fueling advancements in science, literature, law, and philosophy. The shift from oral to written communication set the stage for further technological innovations, including the development of AI.

From manual calculations to AI-driven statistical software: the evolution of mathematical tools

As summarized in Table 1, similar to how the transition from oral traditions to written communication revolutionized the sharing of knowledge, the evolution of mathematical tools - from manual calculations to AI-driven statistical software - represents a significant leap in human progress, demonstrating how technology has greatly enhanced our capacity to process and analyze complex information.

The history of the electronic hand-held calculator began in the mid-1960s when Texas Instruments aimed to promote integrated circuits. In 1966, a team of engineers developed the CAL-TECH, the first portable calculator. In 1970, Texas Instruments partnered with Canon to release the lighter, more marketable Pocketronic [10]. The scientific calculator, a handheld, battery-powered device, is popular for its ability to perform advanced functions such as graph plotting, solving equations, and conducting statistical and matrix operations. Initially developed by Casio in 1985 and later advanced by Texas Instruments in 1995, it revolutionized statistical problem-solving by making tasks previously limited to computers accessible on a portable device. Teachers protested against calculator use in schools in 1988, fearing it would hinder students' ability to learn basic math skills [11].

Alongside this technological progress, early sexist comments depicted calculators as tools primarily for men in professional roles, while women were often seen as using them for domestic tasks such as shopping or managing finances. These stereotypes reflected broader gender inequalities and discrimination in the tech industry, highlighting how societal biases influenced both the development and perception of technology [10].

A study examined the impact of calculator use on secondary school students' numerical skills. The findings revealed that science teachers, regardless of gender, experience, or qualifications, acknowledged both the positive and negative effects of calculators [12]. While calculators were recognized for improving efficiency, problem-solving, and confidence in tackling complex tasks, over-reliance was noted to potentially weaken fundamental computational skills. The study emphasized the importance of using calculators as supplementary tools, ensuring they enhance rather than replace critical mental skills [12].

Prior to the widespread use of computers, statisticians were entirely dependent on paper for computations. Tables (e.g., t-distribution tables, normal distribution tables) were heavily used. The shift from paper-based calculations to statistical software began in earnest in the 1960s and 1970s with the advent of early statistical packages such as SPSS, BMDP, and SAS [13]. Over the following decades, computing power and software sophistication advanced rapidly, allowing statisticians to conduct more complex analyses faster and more accurately. Today, statistical software such as R, Python, SAS, SPSS, and Stata is widely used, and much of the field of statistics is built around the capabilities of these tools [13].

Using calculators and statistical software has both its losses and gains. On the downside, excessive reliance on these tools can diminish basic calculation skills, such as mental math and manual computations, and may lead to a shallow understanding of mathematical concepts [14]. Without a strong grasp of the underlying principles, users might focus on getting quick answers rather than truly understanding the process. Moreover, relying on technology could reduce the development of problem-solving abilities. However, the gains are significant. Calculators and statistical software greatly improve speed and efficiency, allowing for more accurate results and saving valuable time, especially in complex calculations. These tools also offer access to advanced data analysis, pattern recognition, and predictive modeling that would be difficult to achieve manually [13]. When used properly, they can enhance learning by helping students and professionals explore and visualize intricate concepts, ultimately offering deeper insights and empowering more sophisticated analysis.

Convenience and cognitive skills: the impact of the global positioning system (GPS) on navigation

Among the technological shifts outlined in Table 1, GPS technology stands out for how it has redefined human interaction with space and place. Once dependent on maps, memory, and environmental cues, navigation today has become nearly frictionless through GPS-enabled devices, which offer instant directions, real-time traffic updates, and route optimization [15]. However, this convenience comes at a cognitive cost. Researchers have raised concerns that constant reliance on GPS weakens spatial memory and reduces engagement with one’s environment. People no longer need to remember landmarks or street names, which may weaken our ability to navigate without technology [15-17]. The use of GPS reduces reliance on environmental cues, weakening navigational skills, such as map reading, spatial orientation, and memory for landmarks. Although the system offers clear benefits in terms of efficiency and accessibility, its widespread adoption reflects a broader trend: as technologies simplify tasks, they often diminish the cognitive effort once required to perform them. This trade-off raises important questions about the long-term effects of convenience on mental engagement and autonomy [15-18].

Excessive use of GPS navigation has been shown to have a detrimental effect on spatial memory, particularly by diminishing an individual's engagement with their environment. Unlike traditional navigation tools, such as maps, which require active involvement in understanding and orienting oneself to the surroundings, GPS technology offers a more passive experience [17,18]. This reduced engagement leads to a decline in the ability to form cognitive maps, a critical skill that depends on attentively observing landmarks and updating one's position. As individuals rely more on GPS, they become less aware of their surroundings, potentially impairing their ability to navigate independently without technological assistance [17]. Over time, this habit can lead to a dose-dependent decline in spatial memory, as shown in the study, where those with greater GPS reliance experienced more significant deficits in their navigational abilities [18].

Navigating without GPS involves two main strategies, each activating different brain regions. The first is spatial memory, which consists of memorizing landmarks' locations and forming a mental map of the environment. This process heavily depends on the hippocampus and is connected to episodic and relational memory [19,20]. The second strategy, stimulus-response learning, involves memorizing a sequence of actions, such as turning left at specific points. This strategy depends on the caudate nucleus, a brain region also responsible for habit learning, such as acquiring skills like riding a bicycle [15,21]. These two approaches function independently, meaning that damage to one circuit impairs that type of learning while leaving the other intact. However, GPS navigation primarily relies on stimulus-response learning, offering step-by-step instructions that reduce the need for spatial memory and cognitive mapping, leading to less engagement with the environment [22].

This decline in spatial memory is not accompanied by a loss of confidence in one's sense of direction, which suggests that the relationship between GPS use and memory impairment is likely causal. While other navigation methods may engage users more actively, GPS primarily offers step-by-step instructions, minimizing cognitive load and limiting spatial learning. This lack of mental engagement can have broader consequences in real-world scenarios, particularly in regions where traditional navigation skills are essential for safety [15]. For example, Inuit communities rely on environmental cues such as wind currents and snowdrift patterns for navigation, methods that GPS does not capture [15]. If people become too dependent on GPS, they may overlook crucial landmarks and safety considerations, emphasizing the need for future technologies to incorporate more environmental features, such as landmarks, to re-engage users with their surroundings [22].

The Industrial Revolution: fear of job loss

As presented in Table 1, similar to how the advent of writing and calculators revolutionized the way humans learned, stored and shared knowledge, the Industrial Revolution brought about significant changes in manufacturing processes, economic systems, and everyday life.

The Industrial Revolution refers to a major change in industry, technology, and society, shifting economies from agriculture-based to industrialized systems. It involved the introduction of machinery, the use of new energy sources such as steam, and innovations in manufacturing processes, which drastically altered the production of goods and services [1]. This revolution led to the rise of urban centers, as factories became the hubs of economic activity and spurred advancements in transportation and communication [1]. The Industrial Revolution is typically categorized into four distinct phases: the First Industrial Revolution (18th-19th centuries) centered on mechanization and steam power; the Second Industrial Revolution (late 19th to early 20th century), characterized by the use of electricity, mass production, and the assembly line; the Third Industrial Revolution (late 20th century) marked by the rise of computers, automation, and digital technologies; and the Fourth Industrial Revolution (21st century), driven by AI, robotics, IoT, and biotechnology [1]. Each phase has significantly reshaped economies and societies worldwide, continuing to influence contemporary developments.

While driving technological advancements and mass production, the Industrial Revolution also brought significant societal changes, particularly in labor markets. Mechanization and automation replaced many manual jobs, creating widespread job displacement, especially in manufacturing and services [23]. This shift led to fears of mass unemployment and social instability. The Industrial Revolution also amplified global inequities, as the effects of mechanization were unevenly distributed across countries. In developing nations, the influx of low-wage jobs combined with exploitative working conditions, such as child labor and hazardous environments, worsened poverty and entrenched social inequalities [23]. However, it also sparked economic growth and new industries, providing factory work, engineering, and management opportunities. The period saw improvements in workplace safety, with machinery and new safety protocols reducing the risks faced by workers [23].

As automation expanded, the need for updated government policies became evident, addressing income inequality, labor rights, and occupational health and safety. Governments must respond by addressing the growing economic gaps, updating labor laws to protect workers, and creating new frameworks for international cooperation to combat poverty and inequality. Reforms aimed at redistributing wealth, improving working conditions, and ensuring a fairer global economy are crucial for balancing the gains in productivity with the protection of vulnerable workers and communities [23]. New forms of labor representation, particularly for independent and nonstandard workers, will be essential to advocate for fair wages and safe working environments [23].

AI: the latest chapter in the evolution of fear and progress

The ongoing evolution of technology has always been accompanied by a blend of fear and progress. From the introduction of writing to the rise of AI, humanity has consistently grappled with the consequences of new tools that reshape society. Initially, innovations were met with skepticism and resistance, as people feared the loss of control, memory, or even personal identity [2,5]. For example, the advent of writing raised concerns that it would weaken human memory and reduce the need for verbal communication [4]. Similarly, AI today is met with fears surrounding job automation, ethical dilemmas, loss of creativity, and even existential risks [1]. AI today also elicits concern across multiple domains. In the labor market, automation threatens employment in logistics, retail, and transportation, where systems such as Amazon’s warehouse robotics and autonomous delivery trials are reshaping the role of human workers. Ethical dilemmas emerge in areas such as predictive policing and facial recognition, where AI applications risk reinforcing systemic biases and eroding civil liberties. In creative sectors, generative AI tools such as OpenAI’s GPT-4 and image generators such as Midjourney raise questions about intellectual property, artistic authenticity, and the marginalization of human creators. These anxieties highlight the delicate balance between the potential benefits and the unknown consequences of technological advancements. Despite these fears, each technological breakthrough has led to undeniable progress, expanding human capabilities and enhancing communication, knowledge-sharing, and problem-solving. However, the balance between fear and progress remains precarious, as every advancement brings new challenges that test humanity’s ability to adapt.

AI stands at the forefront of this ongoing narrative, representing both the promise of a brighter future and the uncertainty of unforeseen consequences. While AI offers the potential to revolutionize industries, improve healthcare, foster innovation, and increase productivity, it also raises critical ethical, societal, and cognitive concerns [6]. Fears of widespread job displacement and the concentration of power in the hands of a few tech companies dominate the conversation [1]. Additionally, there are anxieties about AI’s impact on human cognition, with worries about the erosion of creativity and critical thinking. However, just as past technologies have disrupted industries and challenged societal norms, AI holds the promise of redefined human potential. It could unlock advancements in healthcare, environmental solutions, and personalized education, offering substantial gains in many areas. Much like previous technological shifts, AI prompts the need for careful examination of how society balances the benefits of innovation with the preservation of core human values [24]. As we continue this technological innovation, AI will undoubtedly shape the next chapter in the evolution of both fear and progress.

Conclusion: fear as a catalyst for thoughtful progress

Fear, often seen as a barrier, can serve as a powerful catalyst for reflection and responsible innovation. Throughout history, each major technological advancement, from writing to GPS, was met with skepticism and ultimately brought societal change. The emergence of AI presents a similarly pivotal moment, one that evokes anticipation and concern. This fear urges us to examine not just the capabilities of these tools, but the values, priorities, and ethical frameworks guiding their development.

To respond responsibly, we must shift from passive observation to active participation. Policymakers have a duty to implement clear and enforceable standards that promote transparency, accountability, and human rights. Educators must prepare future generations with the critical thinking and digital literacy skills needed to engage with AI ethically and constructively. Designers and developers must integrate equity and inclusivity from the outset, ensuring technological progress benefits all communities, not only the privileged.

We cannot afford detachment in shaping the future. Meaningful progress requires engagement informed by historical context and ethical responsibility. When fear is treated not as a deterrent but as a motivator for wise action, it becomes a source of strength. Rather than allowing AI to shape humanity, we must take collective responsibility to define its role in ways that advance justice, human dignity, and shared well-being. The future is not something to passively await; it is something we must actively and courageously create together.
